# Weakened Bayesian Calibration for Tactile Temporal Order Judgment in Individuals with Higher Autistic Traits

**DOI:** 10.1007/s10803-022-05442-0

**Published:** 2022-01-22

**Authors:** Makoto Wada, Yumi Umesawa, Misako Sano, Seiki Tajima, Shinichiro Kumagaya, Makoto Miyazaki

**Affiliations:** 1 Developmental Disorders Section, Department of Rehabilitation for Brain Functions, Research Institute of National Rehabilitation Center for Persons with Disabilities, 4-1, Namiki, Tokorozawa, Saitama 359-8555 Japan; 2 Faculty of Informatics, Shizuoka University, Hamamatsu, Shizuoka 432-8011 Japan; 3 Faculty of Medicine, Kyorin University, Mitaka, Tokyo 181-8611 Japan; 4 Graduate School of Medicine, Nagoya University, Nagoya, Aichi 461-8673 Japan; 5 Department of Child Psychiatry, Hospital of National Rehabilitation Center for Persons with Disabilities, Tokorozawa, Saitama 359-8555 Japan; 6 Research Center for Advanced Science and Technology, The University of Tokyo, Meguro, Tokyo 153-8904 Japan

**Keywords:** Psychophysics, Tactile, Temporal order judgment, Bayesian estimation, Autistic traits, Autism spectrum disorder

## Abstract

Previous psychophysical studies reported a positive aftereffect in tactile temporal order judgments, which can be explained by the Bayesian estimation model (‘Bayesian calibration’). We investigated the relationship between Bayesian calibration and autistic traits in participants with typical development (TD) and autism spectrum disorder (ASD). Bayesian calibration was weakened in TD participants with high autistic traits, consistent with the ‘hypo-priors’ hypothesis for autistic perceptions. The results from the ASD group were generally observed as a continuation of those from the TD groups. Meanwhile, two ASD participants showed irregularly large positive or negative aftereffects. We discussed the mechanisms behind the general results among TD and ASD participants and two particular results among ASD participants based on the Bayesian estimation model.

**Supplementary Information:**

The online version contains supplementary material available at 10.1007/s10803-022-05442-0.

## Introduction

Sensory abnormalities, such as hypersensitivity, are an important symptom of autism spectrum disorder (ASD), which has been primarily characterized as a disability of social communications (American Psychiatric Association, 2013). Certain types of sensory abnormalities can be assessed using psychophysical tasks. One such task is temporal order judgment (TOJ), in which participants judge the orders of two successive sensory stimuli. Using the psychometric function of the TOJ, we can evaluate participants’ temporal resolution and bias in TOJ. For example, ASD participants with higher temporal resolution in tactile TOJ displayed more severe hypersensitivity symptoms (Ide et al., 2019).

TOJ tasks have also revealed the atypical spatiotemporal processing of body representation in individuals with ASD. In typically developing (TD) adults, the crossing of hands increases misreports (i.e., reduces temporal resolution) of tactile TOJ between the hands (Shore et al., 2002; Yamamoto & Kitazawa, 2001). This crossed-hands deficit suggests that the brain takes into account the spatial (extrinsic) locations of the hands before temporally ordering the tactile signals from the respective hands. The degree of crossed-hand deficits was smaller in children with ASD than in those with TD (Wada et al., 2014). This finding suggests a greater reliance on the skin (intrinsic) coordinate in the body representation of children with ASD.

**Fig. 1:**
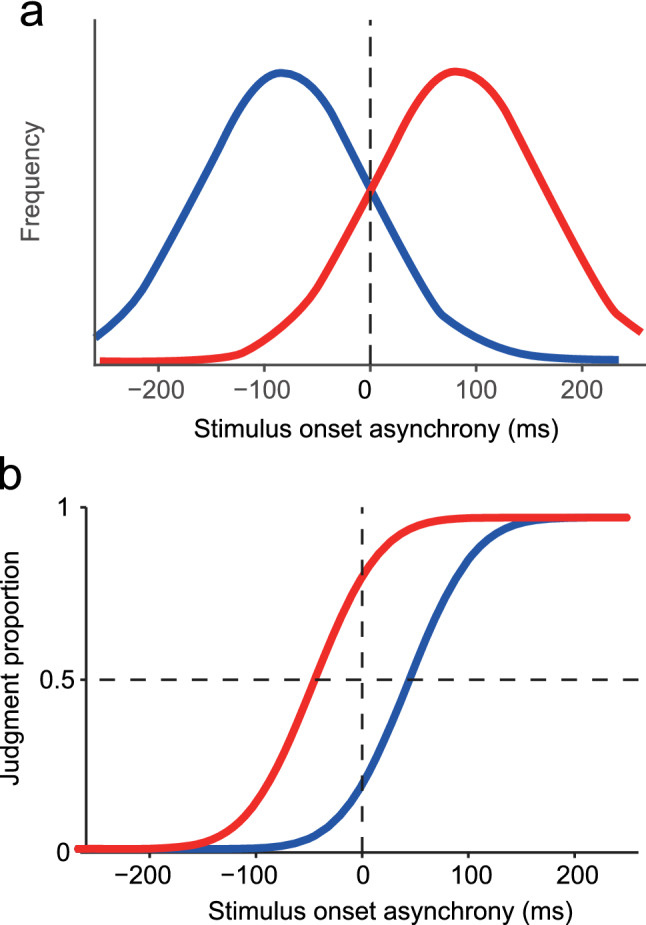
The Bayesian estimation model of temporal order judgment. **a** Examples of biased prior distributions of stimulus onset asynchronies (SOAs): Prior distributions biased to positive (red) and negative (blue) SOAs. **b** The Bayesian estimation model predicts that psychometric functions shift in the opposite directions of the mean SOAs (i.e., most frequent SOAs) in the respective prior distributions. This model predicts a positive aftereffect in tactile temporal order judgment between the hands, as follows (positive SOAs denote that the right hand is stimulated first). For example, after participants frequently experience ‘right then left’ stimuli (i.e., the prior distribution biased to positive SOAs; red line in **a**), their judgments are biased to ‘right then left’ (i.e., the psychometric function shift to negative SOAs; red line in **b**)

Here, we focused on another psychometric parameter, ‘bias.’ Prior experience affects the biases in TOJs (Fujisaki et al., 2004; Hanson et al., 2008; Harrar & Harris, 2008; Miyazaki et al., 2006; Vroomen et al., 2004). In tactile TOJ between the hands, for example, after participants frequently experienced ‘right then left’ stimuli, their judgments were biased to ‘right then left’ at certain rates (Miyazaki et al., 2006; Nagai et al., 2012). This positive aftereffect is accounted for by the optimal Bayesian estimation model (Fig. 1). The Bayesian estimation model assumes that the brain probabilistically optimizes the final estimate of tasks by integrating sensory signals with prior information (Körding & Wolpert, 2004; Körding & Wolpert, 2006). Therefore, the positive aftereffect in tactile TOJ is termed ‘Bayesian calibration’ (Miyazaki et al., 2006).

In the present study, we investigated the relationship between Bayesian calibration and autistic traits in participants with TD and ASD. Pellicano and Burr proposed a Bayesian explanation of the atypical perceptions of autistic people (2012). According to this proposal, sensory abnormalities such as hypersensitivity in people with autism can be explained as attenuated Bayesian priors (‘hypo-priors’). Theoretically, Bayesian priors reduce (i.e., smooth) noisy variations in sensory inputs. Therefore, hypo-priors can cause people with autism to perceive sensory inputs with noise as ‘too real,’ resulting in hypersensitivity. According to the hypo-priors hypothesis, we can predict that Bayesian calibration is weakened among individuals with higher autistic traits.

Hypo-priors have been observed in the interval timing of children with ASD (Karaminis et al., 2016). In contrast, Pell et al. reported that a Bayesian prior was intact in gaze-direction judgment of participants with ASD and TD participants with high autistic traits (Pell et al., 2016). Notably, the results of Pell et al. do not contradict the Bayesian explanation of autistic perception (Pellicano & Burr, 2012). Pellicano and Burr proposed that the Bayesian explanation can systematically account for the diverse atypical behaviors observed in autism, based on whether certain priors are intact or impaired. Thus, our experimental results provide novel evidence to elucidate the Bayesian features behind the atypical perception and behavior of people with autism, regardless of whether the Bayesian calibration is actually weakened among individuals with higher autistic traits.

## Methods

### Participants

Individuals with TD and ASD participated in our experiments (details are described below). Informed consent was obtained from all participants prior to the experiments, and the ethics committees of the National Rehabilitation Center for Persons with Disabilities approved the study. All experiments were performed in accordance with the relevant regulations and guidelines of Japan’s Ministry of Health, Labour, and Welfare.

### TD Participants

Thirty-two paid volunteers participated in the experiments. Two were excluded because their responses could not be fitted to the cumulative Gaussian function (Eq. 1) (*R*^*2*^ of the psychometric function < 0.6). We excluded one additional participant who exhibited inaccurate responses [*σ* = 191 ms > 180 ms (3rd quartile + 2 times the interquartile range)]. Consequently, we conducted the subsequent analyses on data from the remaining 29 participants [15 females, 14 males; 19–43 (26.4 ± 5.77, mean ± standard deviation) years]. There was no such exclusion in previous studies (Miyazaki et al., 2006; Nagai et al., 2012). Some low-reliability fittings would occur because of the smaller number of trials in the present experiments (248 trials/condition) compared to preceding experiments (e.g., 1000 trials/condition). In this study, we implemented fewer trials as an ethical consideration since individuals with ASD are easily fatigued by sensory stimuli.

The autistic traits of each participant were assessed using the Japanese version of the autism spectrum quotient (AQ) score (Wakabayashi et al., 2004) which is based on the English version (Baron-Cohen et al., 2001). The mean AQ score of the TD participants was 16.8 ± 9.22 (mean ± standard deviation; range: 2–35). The general status of the participants was as follows: the handedness of all participants was assessed using the Edinburgh Handedness Inventory [laterality quotient (LQ)] (Oldfield, 1971). There was one left-handed TD participant (LQ = − 73) and one ambidextrous TD participant (LQ = 50); the remaining TD participants were right-handed (LQ = 93.0 ± 9.44; range: 60–100). All participants had normal or corrected-to-normal vision (e.g., glasses, contacts).

### Participants with ASD

Twelve paid volunteers with ASD participated in the experiments. Four were excluded because their responses could not be fitted to the Gaussian cumulative function. Consequently, we conducted subsequent analyses on the remaining eight participants with ASD. These participants were diagnosed by a medical doctor; diagnoses included ASD and pervasive developmental disorders (PDD) (Table 1). Several participants had additional diagnoses of attention-deficit hyperactivity disorder (ADHD) or learning disorders (LD).

**Table 1:** Profiles of participants with ASD

ID	Sex	Age	LQ	AQ	IQ			ADOS-2			Diagnosis	Medication	*σ* (ms)
					Full	Verbal	Non-verbal	Comm	SI	Comm + SI			
#1	M	16	100	26	110	86	140	3	7	10	ASD, ADHD	**–**	28.3
#2	F	37	78	29	100	104	95	3	6	9	PDD, ADHD, LD, Depression	Duloxetine, Zopiclone	76.1
#3	M	35	80	41	136	144	122	3	7	10	ASD, ADHD	Methylphenidate	62.5
#4	M	23	90	24	122	134	103	4	6	10	PDD	–	74.7
#5	M	21	100	32	90	95	91	6	5	11	PDD	–	27.2
#6	M	21	70	36	96	95	98	2	5	7	PDD	Atomoxetine	8.52
#7	M	45	100	34	110	113	103	5	9	14	ASD, Depression	Quetiapine, Clonazepam	48.9
#8	M	27	60	35	140	145	129	3	7	10	ASD, Panic disorder	Aripiprazole, Escitalopram	15.6

*LQ* Laterality Quotient (Edinburgh Inventory); *AQ* Autism-spectrum Quotient; *IQ* Intelligence Quotient; *ADOS*-2 Autism Diagnostic Observation Schedule Component, Second Edition; *Comm*. Communication score (cutoffs: 3/2); *SI* Social Interaction score (cutoffs: 6/4); *Comm* + *SI* total score (communication and social interaction) (cutoffs: 10/7). The cutoffs mentioned in parentheses denote the minimum scores for diagnosing ASD. *PDD* pervasive developmental disorder; *ASD* autism spectrum disorder; *ADHD* attention deficit and hyperactivity disorder; *LD* learning disorder; *σ*, standard deviation in the Gaussian psychometric function (see Eq. 1). Larger (smaller) *σ*s imply lower (higher) sensory temporal resolutions. Each *σ* in this table is the root mean square (*RMS*) of the *σ*s for the left-first biased, right-first biased, and unbiased conditions for each participant

All participants with ASD received the Japanese version (Kuroda & Inada, 2015) of the Autism Diagnostic Observation Schedule Component, Second Edition (ADOS-2) (Lord et al., 2012) and a Japanese version (Fujita et al., 2006) of the Wechsler Adult Intelligence Scale-III (Wechsler, 1997). The ADOS-2 scores were evaluated by an occupational therapist (M. S.) who had a research license for the ADOS-2. Table 1 presents the profiles of each participant with ASD, including any prescribed psychiatric medication.

### Task

**Fig. 2:**
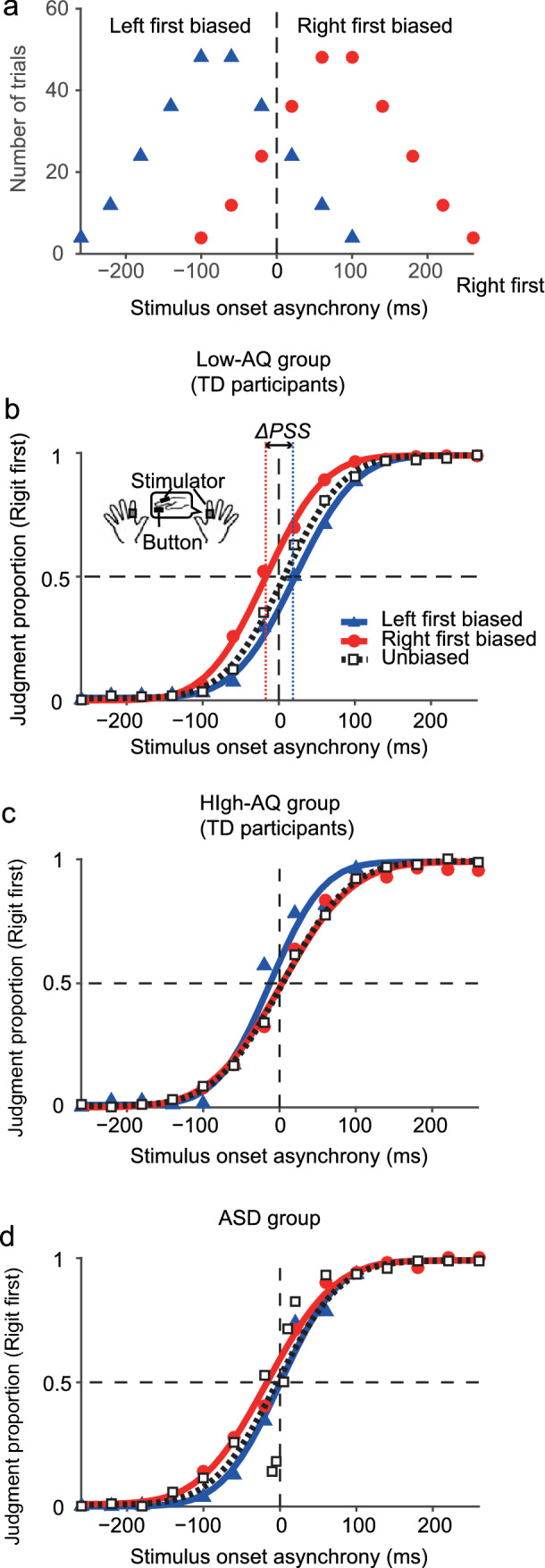
Prior distributions and judgment proportions averaged across participants in the present experiments. **a** Prior distributions for the left-first biased (filled blue triangles) and right-first biased (filled red circles) conditions. The trial numbers (per one participant) are plotted against the stimulus onset asynchronies (SOAs) for each condition. Positive SOAs indicate that the right hand was stimulated first (Right first). The prior distributions had a mean of ± 80 ms and standard deviation of 80 ms. **b** Judgment proportions (Right first) of TD participants in the low-AQ group (AQ < 26, *n* = 22) as a function of SOA. Blue solid, red solid, and black broken curves show the psychometric functions fitted to the judgment proportions of the left-first biased (filled blue triangles), right-first biased (filled red circles), and unbiased (open black squares) conditions, respectively. The points of subjective simultaneities (*PSS*s) were obtained as the SOAs at which the judgment proportions were 0.5. If Bayesian calibration occurs, the difference in the *PSS*s between the left-first and right-first biased conditions (*ΔPSS*) should be larger than zero. **c** Judgment proportions and the fitted psychometric functions of TD participants in the high-AQ group (AQ ≥ 26, *n* = 7). **d** Judgment proportions and the fitted psychometric functions of the ASD group (see Table 1 for their profiles, *n* = 8)

Participants judged the orders of two successive tactile stimuli that were delivered to each hand separately. The participants placed their hands palm down on the desk and received tactile stimuli on the dorsal surface of both index fingers, which were delivered by a computer-controlled tactile stimulator using solenoids (Uchida Denshi, Tokyo, Japan) (see inset in Fig. 2b). To produce each tactile stimulus, a rectangular voltage pulse (11 V, 10 ms) was applied to each solenoid stimulator. The distance between the index fingers was 20 cm. A response button was placed under each index finger pad. During the experiments, participants closed their eyes while white noise (≈ 80 dB) was played through headphones placed over the participants’ ears with earplugs to prevent them from hearing sounds created by the tactile stimuli.

The experiment consisted of three conditions: left-first biased, right-first biased, and unbiased. The order of the conditions was counterbalanced among participants, and the three conditions were conducted on the same day.

In the left-first and right-first biased conditions, stimulus onset asynchronies (SOAs) were sampled from discrete approximations of Gaussian distributions with a mean of ± 80 ms and a standard deviation of 80 ms (Fig. 2a). Positive SOAs indicated that the right hand was stimulated first. The SOA for each trial was randomly assigned from ten possible SOAs ranging from − 260 to 100 ms in the left-first biased condition (− 260, − 220, − 180, − 140, − 100, − 60, − 20, 20, 60, and 100 ms) or from − 100 to 260 ms in the right-first biased condition (− 100, − 60, − 20, 20, 60, 100, 140, 180, 220, and 260 ms) with appearance ratios of 1, 3, 6, 9, 12, 12, 9, 6, 3, and 1 (for a total of 62 trials/epoch), respectively. Each participant experienced four epochs of SOAs for each condition. Therefore, each participant performed 62 × 4 = 248 trials for each condition.

In the unbiased condition, the SOA for each trial was randomly assigned from 14 possible SOAs ranging from − 260 to 260 ms (− 260, − 220, − 180, − 140, − 100, − 60, − 20, 20, 60, 100, 140, 180, 220, and 260 ms) with a uniform distribution (i.e., 14 trials/epoch). In this condition, 18 epochs were repeated, and thus, the total number of trials in this condition was 14 × 18 = 252.

The TOJ task was conducted in a two-alternative forced-choice manner. The participants were instructed to report their judgments by pressing the button on the side that was stimulated first as quickly as possible, as in a previous study using tactile TOJ (Yamamoto & Kitazawa, 2001). The participants were also requested to perform the TOJ task naively without any strategy. They were instructed not to intentionally anticipate the orders to be in line with those from prior trials, and not to intentionally anticipate the orders to be opposed to those in prior trials. For example, in our preliminary experiment, one participant selectively aimed to detect opposite orders to those of prior trials. The participant had attended many psychological experiments and had a preconception that her responses should not be biased towards a specific side. This instruction aimed to prevent potentially biased preconceptions and strategies.

When the reaction time was longer than 5000 ms or when the reaction was earlier than the onset of the second stimulus, we replaced the trial with the same SOA at the end of each condition. No feedback was provided to the participants.

### Data Analysis

TD participants were separated into low-AQ and high-AQ groups. The low-AQ group comprised TD participants with AQ scores of less than 26 (*n* = 22). The high-AQ group comprised TD participants with AQ scores of 26 or more (*n* = 7). This grouping was based on previous evidence that showed an AQ score of ≥ 26 is indicative of the possibility that an individual has ASD (Woodbury-Smith et al., 2005). Participants with ASD (*n* = 8) had diverse backgrounds (i.e., additional diagnoses and medications), as shown in Table 1.

We sorted the response data from each condition based on the SOA to calculate the proportions of the trials in which participants judged the right hand as being stimulated first. The judgment proportion (*p*) can be fitted to a psychometric function using a cumulative Gaussian function (Yamamoto & Kitazawa, 2001):

1 $$p(t)\, = \,(\mathop p\nolimits_{\max } \, - \,p_{\min } )\int\limits_{ - \infty }^{t} {\frac{1}{{\sqrt {2\pi \sigma } }}\exp \left[ {\frac{{ - \left( {\tau - d} \right)^{2} }}{{2\sigma^{2} }}} \right]} \,d\tau \, + \,p_{\min }$$

where *t*, *d,* and *σ* denote the SOA, mean, and standard deviation, respectively, in the fitted Gaussian function. *p*_max_ and *p*_min_ denote the upper and lower asymptotes of the judgment proportion, respectively. In this study, *d*, *σ*, *p*_max_, and *p*_min_ were constrained to − 400 to + 400 ms, 1–1000 ms, 0.99–1.0, and 0.0–0.01, respectively. MATLAB, with the optimization toolbox (MathWorks, Natick, MA, USA), was used for fitting using the maximum likelihood estimation method.

The bias and temporal resolution in the TOJ can be evaluated by the mean and standard deviation of the psychometric function (*d* and *σ* in Eq. 1), respectively. The mean implies the point of subjective simultaneity (*PSS*). The standard deviation implies the temporal uncertainty (i.e., the inverse of the temporal resolution).

Bayesian calibration (Miyazaki et al., 2006) should appear as shifts of the *PSS* in directions opposite to the mean SOAs (peaks in frequency) of prior distributions (Fig. 1a, b). The *PSS* shifts are expressed by Eq. (2) [see Eqs. (1–16) in the Supplementary Methods (https://www.nature.com/articles/nn1712#Sec2) of Miyazaki et al. (2006) for derivation]:

2 $$PSS={ -\left(\frac{{\sigma }_{\mathrm{sensed}}}{{\sigma }_{\mathrm{prior}}}\right)}^{2}{\mu }_{\mathrm{prior}}$$

where *σ*_sensed_ denotes the sensory uncertainty (i.e., the inverse of the sensory temporal resolution) in the TOJ. According to the Bayesian estimation model of the TOJ, *σ*_sensed_ is not affected by prior experience [see Eq. (1), (14), and (15) in the Supplementary Methods of Miyazaki et al. (2006)]. In the present experiments, there was no difference in the *σ* according to differences in prior experience (i.e., left-first biased, right-first biased, or unbiased conditions; *p* = 0.40, see the last paragraph in Results for details), as predicted by the Bayesian estimation model. Therefore, *σ*_sensed_ can be measured as the standard deviation of the psychometric function (*σ* in Eq. 1). *σ*_prior_ and *μ*_prior_ are the standard deviation and mean of the prior distribution, respectively. In our experiments, we used 80 ms for *σ*_prior_ and ± 80 ms for the *μ*_prior_.

We evaluated whether Bayesian calibration occurred using *ΔPSS* (see Fig. 2b). The *ΔPSS* was calculated by subtracting the *PSS* under the right-first biased condition from that under the left-first biased condition. If Bayesian calibration occurs, the *ΔPSS* should be positive.

Moreover, we evaluated the consistency of the observation values of *ΔPSS* with the theoretical predictions using the Bayesian estimation model. The greater positive values of *ΔPSS* imply a higher dependency on the prior but do not always imply an execution of the ‘optimal’ Bayesian estimation. Excessive dependency on prior experience leads to larger errors. To generate an optimal estimation, it is necessary to moderately depend on prior experience according to the degree of sensory temporal resolution of each participant. To evaluate this, we calculated the deviation rate of the observed *ΔPSS* relative to the theoretical *ΔPSS* (%*ΔPSS*_obs−theor_, Eq. 3) for each participant.

When computing the theoretical *ΔPSS*, we used the root mean square (*RMS*) of the *σ* values calculated from the psychometric functions of the left-first biased, right-first biased, and unbiased conditions. Then, we assigned *RMS σ* to *σ*_sensed_ in Eq. (2). This process is reasonable because prior experience has no effect on *σ*_sensed_, as described above. In addition, this averaging process also contributed to stabilizing the *σ*_sensed_ values and the resultant theoretical *ΔPSS* values. For the other parameters in Eq. (2), we assigned 80 ms to *σ*_prior_ and ± 80 ms to the *μ*_prior_. Thus, we obtained a *ΔPSS* value that was theoretically optimal for the sensory temporal resolution of each participant. We then calculated the *%ΔPSS*_obs−theor_ for each participant as:

3 $${\mathrm{\%}\Delta PSS}_{{\text{obs}}-{\text{theor}}}=\left(\frac{{\Delta PSS}_{\text{obs}}-{\Delta PSS}_{\text{theor}}}{{\Delta PSS}_{\text{theor}}}\right) \times 100$$

where *ΔPSS*_obs_ denotes the observed *ΔPSS*, and *ΔPSS*_theor_ denotes the theoretical value*.* The *%ΔPSS*_obs−theor_ is zero when a positive aftereffect occurs, as predicted by the Bayesian estimation model. The *%ΔPSS*_obs−theor_ should be larger than zero when the participant exhibited a larger positive aftereffect relative to the theoretical prediction, whereas it should be smaller than zero when the participant exhibited a smaller aftereffect relative to the theoretical prediction.

## Results

Figure 2a shows the prior distributions for the left-first biased condition (blue triangle) and right-first biased condition (red circle). Figure 2b–d shows the proportions of the trials in which the participants judged ‘the right hand was stimulated first’ as a function of SOA (b: low-AQ group, c: high-AQ group, d: ASD group). The blue, red, and black broken lines in Fig. 2b–d indicate the psychometric functions fitted to the judgment proportions for the left-first biased, right-first biased, and unbiased conditions, respectively.

In Fig. 2b [low-AQ group (TD participants)], the psychometric functions shifted in the opposite directions of the mean SOAs (peaks in frequency) of the respective prior distributions. These shifts of the psychometric functions indicate that a positive aftereffect occurred in response to the prior distributions, which is consistent with the predictions of the Bayesian estimation model (Fig. 1b). In contrast, as shown in Fig. 2c, a positive aftereffect was not observed in the high-AQ group (TD participants), but rather shifted slightly in the same direction. Moreover, a positive aftereffect was also not apparent in the ASD group (Fig. 2d).

**Fig. 3:**
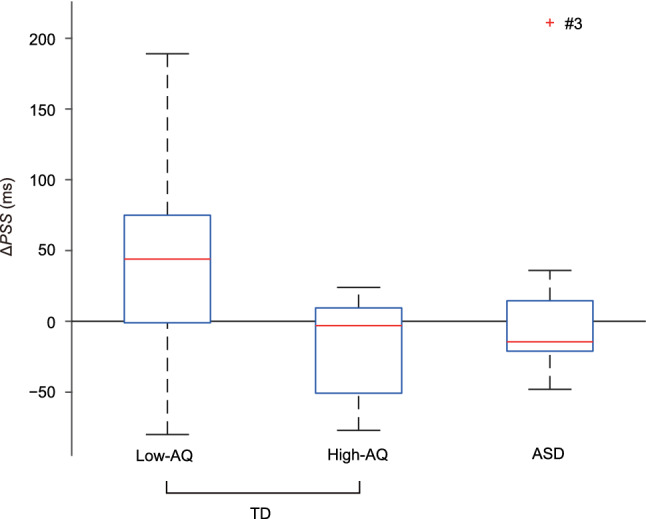
*ΔPSS* across the participants (box-and-whisker plots) in the low-AQ (*n* = 22), high-AQ (*n* = 7), and ASD (*n* = 8) groups. The red line indicates the median in each group, while the upper and lower sides of the blue box indicate the third and first quartiles, respectively. The upper whisker indicates the maximum values within the third quartile + two times the interquartile ranges, and the lower whisker indicates the minimum values within the first quartile − two times the interquartile range. A red cross indicates an outlier greater than the third quartile + two times the interquartile range in the ASD group (ASD participant #3). The mean values ± SEM of *ΔPSSs* were 42.3 ± 12.1 ms for the low-AQ group (*n* = 22), − 17.0 ± 14.6 ms for the high-AQ group (*n* = 7) and 15.0 ± 29.3 ms for the ASD (*n* = 8) groups

Figure 3 shows box-and-whisker plots of the *ΔPSS* values across participants in the low-AQ, high-AQ, and ASD groups. The Kolmogorov–Smirnov test did not reject the normality of *ΔPSS* values across participants for the respective groups (low-AQ, *p* = 0.93; high-AQ, *p* = 0.58; ASD, *p* = 0.21). For the low-AQ group, the *ΔPSS* was significantly greater than zero (*t*_21_ = 3.49, *p* = 0.0022 < 0.05/3, Cohen’s *d* = 0.74, paired *t*-test with the Bonferroni correction). This result is consistent with previous studies that reported a Bayesian calibration in tactile TOJs (Miyazaki et al., 2006; Nagai et al., 2012).

However, the *ΔPSS* was not significantly different from zero for the high-AQ group (*t*_6_ = − 1.17, *p* = 0.29 > 0.05/3, Cohen’s *d* = 0.44). Thus, for the TD participants, a positive aftereffect was evident in the low AQ group, but not in the high AQ group. For the ASD group, *ΔPSS* was not significantly greater than zero (*t*_7_ = 0.51, *p* = 0.62 > 0.05/3, Cohen’s *d* = 0.18). Thus, a positive aftereffect was not observed in the ASD participants, similar to the TD participants in the high-AQ group. These results are consistent with our predictions based on the hypo-priors hypothesis (Pellicano & Burr, 2012).

Moreover, we compared the *ΔPSS* values among groups. Compared to the low-AQ group, the *ΔPSS* was significantly smaller for the high-AQ group (*t*_15.1_ = − 3.13, *p* = 0.0068 < 0.05/3, Cohen’s *d* = 1.02, Welch’s *t*-test with the Bonferroni correction). There was no significant difference in *ΔPSS* between the high-AQ and ASD groups (*t*_10.2_ = − 0.98, *p* = 0.35 > 0.05/3, Cohen’s *d* = 0.49). However, the *ΔPSS* was not significantly different between the low-AQ and ASD groups (*t*_9.51_ = 0.86, *p* = 0.41 > 0.05/3, Cohen’s *d* = 0.42). Notably, one ASD participant (#3) exhibited a large *ΔPSS* [211 ms > 53.7 ms (3rd quartile + 2 times the interquartile range across the ASD participants)]. Excluding the *ΔPSS* for ASD participant #3, the *ΔPSS* was significantly smaller in the ASD group than in the low-AQ group (*t*_23.4_ = − 3.58, *p* = 0.0015 < 0.05/3, Cohen’s *d* = 0.99). As shown in Table 1, ASD participant #3 had an additional diagnosis of ADHD and was prescribed methylphenidate for his ADHD symptoms.

**Fig. 4:**
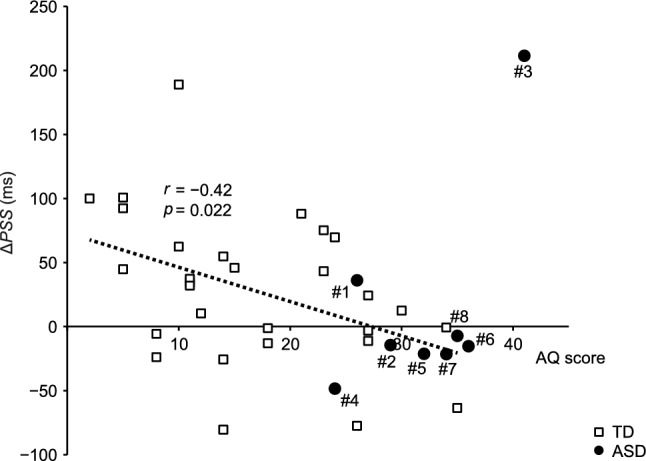
Correlation between the *ΔPSS*s and AQ scores. Black open squares indicate the *ΔPSS*s of TD participants (*n* = 29). The dotted line denotes the regression line between the *ΔPSS*s and AQ scores for the TD participants (slope: − 2.68, intercept: 73.0). Black closed circles indicate the *ΔPSS*s of the participants with ASD (*n* = 8)

Figure 4 shows the *ΔPSS* values of the respective participants plotted as a function of the AQ score. The black open squares denote the *ΔPSS* values of the TD participants, and the dotted line denotes the regression line against the AQ score (slope: − 2.68, intercept: 73.0). A significant negative correlation was observed between the *ΔPSS* and AQ scores among TD participants (*r* = − 0.42, *p* = 0.022, *n* = 29). This result indicates that the positive aftereffect was weakened in TD participants with higher autistic traits.

The black closed circles in Fig. 4 denote the *ΔPSS* values of the participants with ASD. Similar to #3 in Fig. 3, the numbers at the respective *ΔPSS* plots correspond to the ASD participants’ numbers in Table 1. The *ΔPSS* values for the ASD participants generally formed a common distribution with those of the TD participants. However, as also seen in Fig. 3, ASD participant #3 exhibited a large *ΔPSS* value [211 ms > 193 ms (3rd quartile + 2 times the interquartile range across the TD and ASD participants)]. Calculating all values of the TD and ASD participants, the correlation between the *ΔPSS* values and AQ scores was not significant (*r* = − 0.23, *p* = 0.17, *N* = 37). After exclusion of the value of ASD participant #3, a significant negative correlation was observed between the *ΔPSS* and AQ scores (*r* = − 0.49, *p* = 0.0027, *n* = 36).

**Fig. 5:**
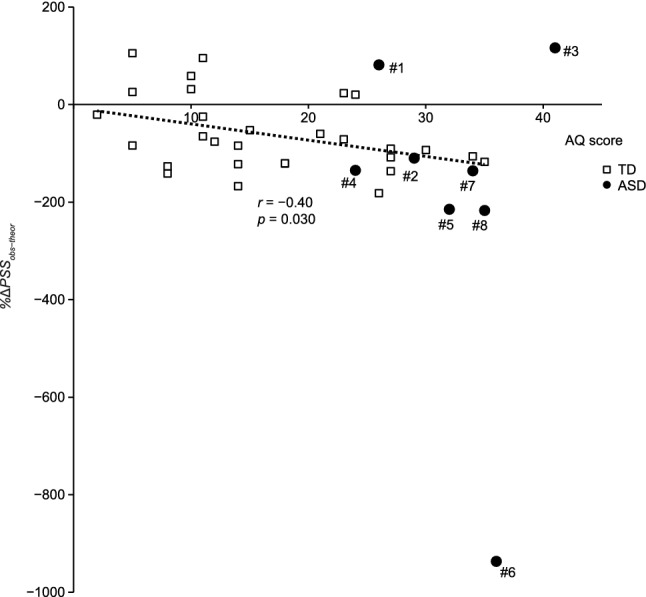
Correlation between the %*ΔPSS*_obs−theor_ values and AQ scores. Black open squares indicate the *ΔPSS*s of TD participants (*n* = 29). The dotted line denotes the regression line between the %*ΔPSS*_obs−theor_ values and AQ scores for the TD participants (slope: − 3.34, intercept: − 6.20%). Black closed circles indicate the *ΔPSS*s of participants with ASD (*n* = 8)

Figure 5 shows the *%ΔPSS*_obs−thoer_ values of the respective participants plotted as a function of the AQ score. The black open squares denote the *%ΔPSS*_obs−thoer_ values of the TD participants, and the dotted line denotes the regression line against the AQ score (slope: − 3.34, intercept: − 6.20%). The *%ΔPSS*_obs−thoer_ values were distributed around zero in the range of AQ scores ≤ 10. However, the *%ΔPSS*_obs−thoer_ values decreased with higher AQ scores, and all values were distributed below zero in the range of AQ scores ≥ 26. There was a significant negative correlation between the *ΔPSS*_obs−thoer_ values and AQ scores among TD participants (*r* = − 0.40, *p* = 0.030, *n* = 29). This result implies that TD participants with higher AQ scores exhibited smaller *ΔPSSs* compared to the theoretical optimal values. That is, the Bayesian calibration was weaker in TD participants with higher autistic traits.

The black closed circles in Fig. 5 denote the *%ΔPSS*_obs−thoer_ values of the participants with ASD. These plots of the ASD participants generally formed a common distribution with the TD participants. Notably, ASD participants #3 and #6 exhibited large positive and negative *%ΔPSS*_obs−thoer_ values, respectively. For ASD participant #3, compared to the *ΔPSS* (Fig. 4)*,* the positive amplitude was moderate (116% < 191% [3rd quartile + 2 times the interquartile range across the TD and ASD participants]); however, the value of 116% deviated by 259% from that of the regression line (− 143%) at the AQ score of 41 for ASD participant #3 (Table 1). ASD participant #6 exhibited an extremely large negative value (− 937% < − 338% [1st quartile − 2 times the interquartile range across the TD and ASD participants]). As shown in Table 1, ASD participant #6 was diagnosed with PDD and had been prescribed atomoxetine. Even though the values of participants #3 and #6 were included, the negative correlation between the *ΔPSS*_obs−thoer_ values and AQ scores was significant among the TD and ASD participants (*r* = − 0.36, *p* = 0.029, *N* = 37). Moreover, after exclusion of the values of participants #3 and #6, the negative correlation between the *ΔPSS*_obs−thoer_ values and AQ scores remained significant (*r* = − 0.47, *p* = 0.0044, *n* = 35).

For another psychometric measurement, *σ*, there was no significant association between the AQ score and ASD diagnosis. Table 2 shows the *σ* values across the participants for the left-first biased, right-first biased, and unbiased conditions in the low-AQ, high-AQ, and ASD groups. A two-way analysis of variance (ANOVA) on *σ* with condition (within participants: left-first biased, right-first biased, and unbiased conditions) and group (between participants: low-AQ, high-AQ, and ASD groups) revealed no significant main effect of condition [*F* (1.49, 50.69) = 0.87, *p* = 0.40, partial *η*^*2*^ = 0.025], group [*F *(2, 34) = 1.38, *p* = 0.26, partial *η*^*2*^ = 0.075] and their interaction [*F *(2.98, 50.69) = 1.56, *p* = 0.21, partial *η*^*2*^ = 0.084]; In the ANOVA on *σ*, the degrees of freedom were adjusted using Greenhouse–Geisser’s ε according to Mendoza’s multisample sphericity test (*p* = 0.0055). In addition, there was no significant correlation between the *σ*s and AQ scores across TD and ASD participants (left-first biased condition: *r* = − 0.28, *p* = 0.096; right-first biased condition: *r* = 0.21, *p* = 0.21; unbiased condition: *r* = − 0.23, *p* = 0.18; *N* = 37). In addition, there was no significant correlation between the *σ*s and AQ scores among TD participants (left-first biased condition: *r* = − 0.10, *p* = 0.61; right-first biased condition: *r* = 0.35, *p* = 0.062; unbiased condition: *r* = − 0.00065, *p* = 1.0; *n* = 29). Similar results were obtained when excluding the *σ* value of ASD participant #3 (Supplementary Table S1) or those of ASD participants #3 and #6 (Supplementary Table S2).

**Table 2:** *σ* values across the participants (mean ± SEM) for the left-first biased, right-first biased and unbiased conditions in the low-AQ, high-AQ and ASD groups

	*σ* (ms)		
	Left-first	Right-first	Unbiased
Low-AQ group (TD participants)	61.9 ± 8.44	50.1 ± 7.64	57.1 ± 5.75
High-AQ group (TD participants)	54.7 ± 11.5	73.6 ± 19.5	60.7 ± 15.7
ASD group	35.9 ± 7.75	52.6 ± 13.2	34.2 ± 8.15

*σ* is calculated as the standard deviation of the psychometric function (see Eq. 1)

## Discussion

In the present study, we investigated the relationship between autistic traits and the effects of prior experience on tactile TOJ. Previous psychophysical studies have reported a positive aftereffect in tactile TOJs, which can be accounted for by the Bayesian estimation model (Bayesian calibration) (Miyazaki et al., 2006; Nagai et al., 2012; Yamamoto et al., 2012). As a result, a positive aftereffect in tactile TOJ was observed among TD participants with lower AQ scores, as predicted by the Bayesian estimation model. However, the positive aftereffect decreased in those with higher AQ scores. That is, Bayesian calibration was weakened for TD participants with higher autistic traits.

The results from the ASD participants were generally observed to be a continuation of those from the TD group. Bayesian calibration was not observed among most participants with ASD, along with their high AQ scores. Meanwhile, we also found irregular results in two participants with ASD (#3 and #6). Participants with ASD in our study had diverse backgrounds regarding additional diagnoses and medications (Table 1). The irregular results suggest that TOJ is also affected by other diseases and/or medications other than ASD.

In the following sections, we discuss the mechanisms behind the general results among TD and ASD participants, and the two particular results among ASD participants, based on the Bayesian explanations for autistic perceptions (Pellicano & Burr, 2012).

### Weakened Bayesian Calibration is Consistent with the ‘Hypo-priors’ Hypothesis

In our experiment, Bayesian calibration was weakened in participants with higher autistic traits. According to the Bayesian estimation model (Körding & Wolpert, 2004; Miyazaki et al., 2006; Pellicano & Burr, 2012), there are two possible mechanisms for the result. The first is ‘hypo-priors,’ which is an impairment of learning and/or the utilization of prior distributions leading to weaker Bayesian calibration in participants with higher autistic traits. The second is ‘hyper-sensors,’ in which a higher sensory temporal resolution leads to weaker Bayesian calibration in participants with higher autistic traits. Our results do not support the latter hypothesis. This is due to the lack of a significant difference for the *σ* values among groups, and that there was no significant correlation between the *σ* values and AQ scores (Table 2, Supplementary Tables S1 and S2). Moreover, participants with higher AQ scores exhibited smaller *%ΔPSS*_obs−thoer_ values (i.e., weaker Bayesian calibration), each of which was adjusted by the *σ* value of each participant (Figure 5). Accordingly, the series of our results suggest that the weakened Bayesian calibration observed herein is attributable to the impairment of learning and/or utilization of the prior distributions.

Thus, our results are consistent with the ‘hypo-priors’ hypothesis (Pellicano & Burr, 2012). Hypo-priors have also been observed in the interval timing of children with autism (Karaminis et al., 2016). Interval-timing responses are biased to the mean of the target time intervals (central tendency effect), which can be accounted for by the Bayesian estimation model (Jazayeri & Shadlen, 2010; Miyazaki et al., 2005; Roach et al., 2017). Karaminis et al. reported that in children with autism, the central tendency was much less than that predicted by the theoretical model that took into account the sensory temporal resolution of each participant. Previous findings and our results suggest that hypo-priors generally occur during human sensorimotor processing. Furthermore, we infer that hypo-priors may also be observed in motor controls. Psychophysical studies using motor control tasks first reported that the human brain generates a Bayesian estimation by learning the prior distribution (Körding & Wolpert, 2004; Körding et al., 2004). Moreover, an fMRI study suggested that tactile TOJs share neural bases with motor controls (Miyazaki et al., 2016). Individuals with ASD frequently exhibit developmental coordination disorder (Cacola et al., 2017), some of which may be explained by impairments in the learning of Bayesian priors.

### Similarities and Differences with Previous studies Regarding Audiovisual Lag Adaptation

Several psychophysical studies also reported hypo-priors in audiovisual lag adaptation (or called audiovisual temporal recalibration) (Noel et al., 2017; Stevenson et al., 2017; Turi et al., 2016), although they did not show a mathematical model to specifically explain lag adaptation. Lag adaptation has been observed as a negative aftereffect in audiovisual simultaneity judgment (SJ) (Fujisaki et al., 2004) and TOJs (Hanson et al., 2008; Miyazaki et al., 2006; Vroomen et al., 2004; Yamamoto et al., 2012). The negative aftereffects in SJ and TOJ suggest that after repeated exposure to audiovisual stimuli with a lag, participants eventually perceived the repeated lag to be shorter than that in reality. Lag adaptation is considered to reflect brain adaptation, which compensates for differences in physical and neuronal conduction times between audio and visual signals (Fujisaki et al., 2004). Furthermore, Van der Burg demonstrated that a similar negative aftereffect occurred in response to only one prior experience (rapid lag adaptation) (Van der Burg et al., 2013). Recent studies have reported impairments in the rapid type of lag adaptation in participants with ASD (Noel et al., 2017; Turi et al., 2016).

A more recent study (Stevenson et al., 2017) reported that long-term lag adaptation (Fujisaki et al., 2004) was weakened in TD participants with higher scores of attention to detail (subscale of AQ score); however, there was no correlation between the total AQ score and other subscales. In the present experiments, the *ΔPSS* (amplitude of positive aftereffect) exhibited a significant negative correlation with the total AQ score in the TD participants (Fig. 4). Moreover, *ΔPSS* had significant (*p* < 0.05) or marginally significant (*p* < 0.1) negative correlations with the subscales, including attention to detail, with the exception of social skills and imagination (Supplementary Table S3). In addition, after including the ASD participants (Supplementary Tables S4–S6), similar results were observed when excluding ASD participant #3 (Supplementary Table S5) or participants #3 and #6 (Supplementary Table S6). The difference between our results and those of Stevenson et al. suggests that audiovisual lag adaptation and tactile Bayesian calibration reflect distinctive features in autistic spectrum conditions.

### Particular Cases Among Participants with ASD

We should also note two participants with ASD (#3 and #6) who displayed particular results. ASD participant #3 exhibited an irregularly large positive aftereffect. Notably, participant #3 had an additional diagnosis of ADHD and was prescribed methylphenidate (Table 1). Methylphenidate is a norepinephrine and dopamine reuptake inhibitor (Madras et al., 2005). A dose of methylphenidate increased the learning rate in a probabilistic learning task in healthy human participants (Howlett et al., 2017). The increase in learning rate implies that the participants quickly adjusted their responses. In theory (Howlett et al., 2017; Yu and Huang, 2014), this strategy is optimal for tasks with high volatility, but is not always optimal for static or coherent tasks, such as our TOJ, which is systematically biased to a specific order. Notably, a positive aftereffect itself should appear even when TOJ was biased to the orders in recent prior trials, although it did not lead to optimal judgment in our task. Methylphenidate may have increased the learning rate of participant #3, resulting in an excessive bias to the priors.

ASD participant #6 revealed an irregularly large negative deviation in his aftereffect relative to the theoretical prediction (Fig. 5). Participant #6 did not display a prominent irregular value in the aftereffect (#6 in Fig. 4). This apparent discrepancy was due to his irregularly high sensory temporal resolution (i.e., extremely small *σ*: 8.52 ms, see Table 1). Such extremely high sensory temporal resolution implies that the participant has an extremely high information density per unit of time. In such participants, if a perceptual change occurred in the TOJ, it appeared small in the observed *ΔPSS* value. Since we used the *%ΔPSS*_obs−thoer_, each of which value was standardized by the *σ* value of each participant, we detected the irregularly large negative aftereffect of participant #6 relative to their extremely high sensory temporal resolution. Notably, participant #6 was diagnosed with PDD and prescribed atomoxetine (Table 1). A psychopharmacological study in mice reported that an acute administration of atomoxetine improved temporal precision (Balci et al., 2008). Thus, the small *σ* of participant #6 may have been due to atomoxetine. Moreover, atomoxetine is a norepinephrine reuptake inhibitor (Wong et al., 1982). Therefore, atomoxetine may also increase learning rates. However, at this stage, it is unclear whether atomoxetine caused a negative aftereffect instead of a positive aftereffect, even if atomoxetine was truly involved. Our results may suggest a contrasting pharmacological effect on perceptual/cognitive adaptations between methylphenidate and atomoxetine; however, more pharmacological evidence is needed to ascertain our suggestions.

### Limitations, Perspectives, and a Note on the Present Study

As described above, our results were discussed based on Bayesian explanations for autistic perceptions (Pellicano & Burr, 2012). However, there are limitations to the present study. First, the number of participants with ASD was small (*n* = 8). The results of the ASD participants suggested not only generality along with those of TD participants but also the particularity of certain ASD participants (#3, #6). Although we discussed the possible mechanisms and implications for the particular results of the two ASD participants based on their medications, each explanation was only an ex-post speculative hypothesis based on only one case. The relationship between their behavior and medications may just be due to chance. Large-sample and interventional studies are needed to test these hypotheses. Our hypotheses may be rejected in future studies. However, repetitions of such hypothesis testing will clarify the mechanisms to systematically account for both general and diverse aspects of autistic perception and behaviors.

Compared to the ASD participants, the present study showed more coherent results for TD participants with a larger sample size (*n* = 29). Here, we should note that if a participant without an ASD diagnosis shows a small or no Bayesian calibration, it does not always imply that the participant has ASD. Although the AQ score is a reliable index to evaluate autistic traits, TD individuals with high AQ scores are not always diagnosed with ASD (Wakabayashi et al., 2004). Our results provide evidence to support the Bayesian explanation of autistic perception and behavior (Pellicano & Burr, 2012), but we should not automatically apply our results to the diagnosis of ASD.

## Supplementary Information

Below is the link to the electronic supplementary material.

Supplementary file1 (DOCX 96 KB)

## Data Availability

The datasets used and/or analyzed in the current study are available from the corresponding author upon reasonable request.
