# Supplementary material for: Weakened Bayesian Calibration for Tactile Temporal Order Judgment in Individuals with Higher Autistic Traits
Source: J Autism Dev Disord. 2022 Jan 22;53(1):378–89. doi: 10.1007/s10803-022-05442-0 (PMC9889458; doi:10.1007/s10803-022-05442-0)
Supplement: Supplementary file 1 — Supplementary file1 (DOCX 96 KB) [file 10803_2022_5442_MOESM1_ESM.docx]

|  |  |  |  |  |  |  |  |  |  |  |  |  |
| --- | --- | --- | --- | --- | --- | --- | --- | --- | --- | --- | --- | --- |
|  | ***σ* (ms)** | | | | | | | | | | |  |
|  | **Left-first** | | |  | **Right-first** | | |  | **Unbiased** | | |  |
|  |  |  |  |  |  |  |  |  |  |  |  |  |
| Low-AQ group (TD participants) | 61.9 | ± | 8.44 |  | 50.1 | ± | 7.64 |  | 57.1 | ± | 5.75 |  |
|  |  |  |  |  |  |  |  |  |  |  |  |  |
| High-AQ group (TD participants) | 54.7 | ± | 11.5 |  | 73.6 | ± | 19.5 |  | 60.7 | ± | 15.7 |  |
|  |  |  |  |  |  |  |  |  |  |  |  |  |
| ASD group | 38.1 | ± | 8.60 |  | 47.0 | ± | 13.8 |  | 31.4 | ± | 8.84 |  |
|  |  |  |  |  |  |  |  |  |  |  |  |  |
|  |  |  |  |  |  |  |  |  |  |  |  |  |

Supplementary Table S1: *σ* values across the participants (mean ± SEM) for the left-first biased, right-first biased, and unbiased conditions in the low-AQ, high-AQ, and ASD groups (without ASD participant #3). A two-way analysis of variance (ANOVA) on *σ* with condition (within participants: left-first biased, right-first biased and unbiased conditions) and group (between participants: low-AQ, high-AQ, and ASD) revealed no significant main effect of condition (*F*(1.5,49.48) = 0.54, *p* = 0.54, partial *η^2^* = 0.016), group (*F*(2, 33) = 1.51, *p* = 0.23, partial *η^2^* = 0.084) and its interaction (*F*(3, 49.48) = 1.26, *p* = 0.30, partial *η^2^* = 0.071). In the ANOVA for *σ*, the degrees of freedom were adjusted using Greenhouse-Geisser’s ε according to Mendoza's multisample sphericity test (*p* = 0.0006). There was no significant correlation between the *σ*s and AQ scores across the TD and ASD participants (left-first biased condition: *r* = −0.24, *p* = 0.16; right-first biased condition: *r* = 0.17, *p* = 0.32; unbiased condition: *r* = −0.24, *p* = 0.15; *n* = 36)*. .*

|  |  |  |  |  |  |  |  |  |  |  |  |  |
| --- | --- | --- | --- | --- | --- | --- | --- | --- | --- | --- | --- | --- |
|  | ***σ* (ms)** | | | | | | | | | | |  |
|  | **Left-first** | | |  | **Right-first** | | |  | **Unbiased** | | |  |
|  |  |  |  |  |  |  |  |  |  |  |  |  |
| Low-AQ group (TD participants) | 61.9 | ± | 8.44 |  | 50.1 | ± | 7.64 |  | 57.1 | ± | 5.75 |  |
|  |  |  |  |  |  |  |  |  |  |  |  |  |
| High-AQ group (TD participants) | 54.7 | ± | 11.5 |  | 73.6 | ± | 19.5 |  | 60.7 | ± | 15.7 |  |
|  |  |  |  |  |  |  |  |  |  |  |  |  |
| ASD group | 42.4 | ± | 8.78 |  | 53.8 | ± | 14.2 |  | 35.7 | ± | 9.17 |  |
|  |  |  |  |  |  |  |  |  |  |  |  |  |
|  |  |  |  |  |  |  |  |  |  |  |  |  |

Supplementary Table S2: *σ* values across the participants (mean ± SEM) for the left-first biased, right-first biased, and unbiased conditions in the low-AQ, high-AQ, and ASD groups (without ASD participants #3 and #6). A two-way analysis of variance (ANOVA) on *σ* with condition (within participants: left-first biased, right-first biased and unbiased conditions) and group (between participants: low-AQ, high-AQ, and ASD) revealed no significant main effect of condition (*F*(1.5,48.01) = 0.62, *p* = 0.50, partial *η^2^* = 0.019), group (*F*(2, 32) = 0.81, *p* = 0.45, partial *η^2^* = 0.048) and its interaction (*F*(3, 48.01) = 1.29, *p* = 0.29, partial *η^2^* = 0.075); In the ANOVA on *σ*, the degrees of freedom were adjusted using Greenhouse-Geisser’s ε according to Mendoza's multisample sphericity test (*p* = 0.0016). There was no significant correlation between the *σ*s and AQ scores across the TD and ASD participants (left-first biased condition: *r* = −0.19, *p* = 0.26; right-first biased condition: *r* = 0.24, *p* = 0.16; unbiased condition: *r* = −0.18, *p* = 0.29; *n* = 35)*. .*

|  |  |  |  |  |  |  |
| --- | --- | --- | --- | --- | --- | --- |
|  | **Total AQ** | **Social skill** | **Attention switching** | **Attention to detail** | **Communication** | **Imagination** |
|  |  |  |  |  |  |  |
| ***r*** | −0.42 | −0.21 | −0.35 | −0.34 | −0.39 | −0.24 |
| ***p*** | 0.022 | 0.28 | 0.059 | 0.071 | 0.038 | 0.20 |
|  |  |  |  |  |  |  |
|  |  |  |  |  |  |  |

Supplementary Table S3: Correlation between the *ΔPSS* values and AQ subscale scores for TD participants (*n* = 29).

|  |  |  |  |  |  |  |
| --- | --- | --- | --- | --- | --- | --- |
|  | **Total AQ** | **Social skill** | **Attention switching** | **Attention to detail** | **Communication** | **Imagination** |
|  |  |  |  |  |  |  |
| ***r*** | −0.23 | −0.16 | −0.24 | −0.24 | −0.25 | −0.013 |
| ***p*** | 0.17 | 0.36 | 0.15 | 0.15 | 0.14 | 0.94 |
|  |  |  |  |  |  |  |
|  |  |  |  |  |  |  |

Supplementary Table S4: Correlation between the *ΔPSS* values and AQ subscale scores across TD and ASD participants (*N* = 37).

|  |  |  |  |  |  |  |
| --- | --- | --- | --- | --- | --- | --- |
|  | **Total AQ** | **Social skill** | **Attention switching** | **Attention to detail** | **Communication** | **Imagination** |
|  |  |  |  |  |  |  |
| ***r*** | −0.49 | −0.32 | −0.46 | −0.35 | −0.47 | −0.24 |
| ***p*** | 0.0027 | 0.059 | 0.0053 | 0.034 | 0.0041 | 0.17 |
|  |  |  |  |  |  |  |
|  |  |  |  |  |  |  |

Supplementary Table S5: Correlation between the *ΔPSS* values and subscales of the AQ scores across TD and ASD participants (without ASD participants #3; *n* = 36).

|  |  |  |  |  |  |  |
| --- | --- | --- | --- | --- | --- | --- |
|  | **Total AQ** | **Social skill** | **Attention switching** | **Attention to detail** | **Communication** | **Imagination** |
|  |  |  |  |  |  |  |
| ***r*** | −0.48 | −0.31 | −0.45 | −0.34 | −0.46 | −0.22 |
| ***p*** | 0.0037 | 0.072 | 0.0074 | 0.045 | 0.0057 | 0.20 |
|  |  |  |  |  |  |  |
|  |  |  |  |  |  |  |

Supplementary Table S6: Correlation between the *ΔPSS* values and AQ subscale scores across the TD and ASD participants (without ASD participants #3 and #6; *n* = 35).
